# Socioeconomic determinants and awareness of excise tax on soft drinks in relation to nutritional status among adults in Al-Ahsa Province, Saudi Arabia

**DOI:** 10.3389/fpubh.2026.1830296

**Published:** 2026-10-20

**Authors:** Randah M. Alqurashi, Kawthar A. Alanezi, Samar M. Abdalla

**Affiliations:** 1 Department of Food and Nutrition, College of Agriculture and Food Sciences, King Faisal University, Hofuf, Al-Ahsa, Saudi Arabia; 2 Department of Agribusiness and Consumer Sciences, College of Agricultural and Food Sciences, King Faisal University, Hofuf, Al-Ahsa, Saudi Arabia

**Keywords:** BMI, excise tax, nutritional status, soft drink consumption, tax awareness

## Abstract

**Background:**

Soft drinks are a major contributor to overweight, obesity, and non-communicable diseases. In response, Saudi Arabia introduced an excise tax on soft drinks in 2017. However, evidence regarding public awareness of this tax and its association with socioeconomic factors, consumption behaviors, and nutritional status remains limited.

**Aim:**

This study aimed to examine the association between socioeconomic determinants and awareness of the excise tax on soft drinks in relation to nutritional status among adults in Al-Ahsa Province, Saudi Arabia.

**Methods:**

A cross-sectional survey was conducted between February and March 2023 among adults aged 18–45 years in Al-Ahsa Governorate. Data were collected using a validated electronic questionnaire covering socioeconomic characteristics, soft drink consumption, excise tax awareness, and anthropometric measures. Body mass index (BMI) was calculated using self-reported height and weight. Data analysis included descriptive statistics, chi-square tests, independent *t*-tests, ANOVA, and Spearman correlation coefficients.

**Results:**

A total of 370 participants were included. More than half of respondents (52.7%) were unaware of the excise tax on soft drinks, and 64.9% lacked knowledge of its underlying health rationale. Tax awareness was significantly associated with education level (*p* = 0.02), additional income (*p* = 0.02), gender (*p* = 0.01), and age (*p* < 0.10). While tax awareness was not significantly associated with current soft drink consumption, higher awareness was significantly correlated with reduced soft drink intake (*r* = 0.213, *p* < 0.01), increased consumption of alternative beverages (*r* = 0.117, *p* = 0.02), and BMI classification (*p* = 0.02). However, the finding of analysis of covariance (ANCOVA) found the association between awareness of selective excise taxes and BMI was insignificant (*p* = 0.86) adjusted for selected demographic, social, and economic variables. This implies that awareness of the excise tax is not related to BMI in the context of controlling demographic and socioeconomic variables for this population.

**Conclusion:**

Awareness of the excise tax on soft drinks varies across socioeconomic groups and is positively associated with healthier beverage choices. However, after controlling demographic and socioeconomic characteristics, awareness of the excise tax was not significantly associated with BMI in this population. Thus, enhancing public awareness and education about the purpose and health benefits of the excise tax may strengthen its effectiveness as a public health intervention in Saudi Arabia.

## 1 Introduction

Overweight and obesity have become major global public health concerns, with prevalence increasing rapidly across countries at different stages of economic development (1). Excess body weight is a well-established risk factor for numerous non-communicable diseases (NCDs), including type 2 diabetes mellitus, cardiovascular diseases, hypertension, and certain cancers (2–5). The growing burden of obesity-related conditions poses significant challenges to healthcare systems and highlights the need for effective population-level strategies aimed at improving dietary behaviors and nutritional status (6).

Among dietary risk factors, the consumption of sugar-sweetened beverages (SSBs), particularly soft drinks, has been consistently linked to excessive energy intake, weight gain, and adverse metabolic outcomes (7, 8). Soft drinks are characterized by high levels of added sugars, low nutritional value, and limited satiety, which may promote increased caloric intake without compensatory reductions in food consumption (9). Extensive epidemiological evidence, including systematic reviews and meta-analyses, demonstrates a positive association between frequent SSB consumption and obesity, metabolic syndrome, and type 2 diabetes (10, 11). Consequently, reducing SSB intake has become a central objective of public health nutrition policies (12). In response to the rising prevalence of obesity and diet-related NCDs, fiscal policies such as excise taxes on sugar-sweetened beverages have been increasingly adopted worldwide (13). These taxes aim to discourage unhealthy consumption by increasing product prices and to encourage substitution toward healthier beverage options, including water and unsweetened drinks (14). Empirical evidence from countries such as Mexico, the United Kingdom, and Spain suggests that SSB taxation is associated with reduced purchases and consumption of taxed beverages, alongside increased consumption of untaxed alternatives (15, 16). Such findings support excise taxation as a promising population-level strategy for improving dietary behaviors (17).

However, the effectiveness of excise taxes varies across populations and is influenced by socioeconomic determinants, including education level, income, age, and gender (18, 19). These factors shape individuals' dietary choices, health literacy, and responsiveness to price-based interventions (20). Studies have shown that individuals with higher educational attainment and income levels tend to be more aware of the health risks associated with SSB consumption and more responsive to price signals, whereas lower socioeconomic groups may face barriers related to information access and behavioral change (18, 21, 22).

In addition to socioeconomic factors, public awareness and understanding of excise taxes play a critical role in determining their behavioral impact. Awareness of the existence of the tax, as well as understanding its health-related rationale, may reinforce price signals and increase the likelihood of behavior modification. Evidence indicates that individuals who perceive SSB taxes as health-motivated are more likely to support such policies and reduce their consumption of taxed products (23). Conversely, limited awareness or misinterpretation of tax objectives may weaken the effectiveness of fiscal interventions.

Saudi Arabia has experienced a rapid nutrition and lifestyle transition over recent decades, characterized by increased consumption of processed foods and sugar-sweetened beverages and declining physical activity levels. These shifts have contributed to a high prevalence of overweight and obesity among adults, raising concerns about the long-term burden of NCDs (37). In response, Saudi Arabia introduced an excise tax on soft drinks and energy drinks in 2017 as part of broader national health reforms aligned with Vision 2030. Despite the implementation of this policy, evidence regarding public awareness of the excise tax and its association with consumption behaviors and nutritional outcomes remains limited.

Recent studies in Saudi Arabia have emphasized the role of health awareness in shaping dietary behaviors. Alqurashi et al. (24) reported that higher levels of health awareness related to soft drinks were associated with improved nutritional outcomes among adults, suggesting that knowledge and perception are important determinants of beverage choices. Nevertheless, most existing research has focused on general health awareness rather than specific awareness of excise taxation policies, and few studies have examined how socioeconomic factors interact with tax awareness to influence consumption behaviors and nutritional status.

The study aims to assess the association between socioeconomic determinants and awareness of the excise tax on soft drinks in relation to nutritional status among adults in Al-Ahsa Province, Saudi Arabia. Findings from this study may contribute to a better understanding of how fiscal health policies and social determinants intersect, and may inform future nutrition education, policy communication strategies, and targeted interventions to improve adult nutritional health.

## 2 Materials and methods

### 2.1 The study sample

The research was performed in Al-Ahsa Governorate, Kingdom of Saudi Arabia (KSA). The primary data was gathered from February 9, 2023, to March 23, 2023. A convenience sample was used, and the sample was determined using the Stephen Thompson equation (34), based on the total population of about 1,104,267 individuals in Al-Ahsa Governorate (36). The Stephen Thompson equation is delineated below


$$\begin{array}{l} n=\frac{N\times p\;\left(1-p\right)}{\left[\left[N-1\times \left(\frac{d^{2}}{z^{2}}\right)\right]+p\;\left(1-p\right)\right]} \end{array}$$

where:

*N* = community size (The community size of Al-Ahsa is estimated at 1,104,267 inhabitants (36).

*z* = the normative degree corresponding to the level of significance is 0.95 and is equal to 1.96

*d* = error ratio and is equal to 0.05

*P* = the ratio of property availability and neutral and is equal to 0.50


$$\begin{array}{l} n=\frac{1,104,267\times 0.50\;\left(1-0.50\right)}{\left[\left[1,104,267-1\times \left(\frac{{\left(0.05\right)}^{2}}{{\left(1.96\right)}^{2}}\right)\right]+0.50\;\left(1-0.50\right)\right]} \end{array}$$

Consequently, the minimum sample size selected is about 384 participants. Nonetheless, A total of 419 participants was primarily collected through convenience sample method to obtain complete and accurate data. However, 49 participants were excluded due to incomplete or missing necessary data, resulting in a final study sample of 370 participants (24).

### 2.2 Data collection

The data was gathered by survey methods utilizing an electronic questionnaire aimed at individuals aged 18–45 in Al-Ahsa Governorate. The questionnaire comprises three fundamental sections: the initial portion encompasses personal information, including demographic, social, and economic characteristics of the participants and encompasses the consumption of soft drinks (SDs). The second section denotes awareness and understanding pertaining to the taxation imposed on SDs. The second section denotes awareness and understanding pertaining to the taxation imposed on SDs. This section included numerous inquiries concerning participants' perceptions of awareness and knowledge regarding the SDs tax. These are:

- Saudi society in general consumes more soft drinks.
- Knowledge of the imposition of excise tax on soft drinks in the Kingdom of Saudi Arabia.
- The impact of excise taxation on the reduction in soft drink consumption.
- The rate of reduction in soft drink consumption resulting from the imposition of excise tax.
- Awareness of the reasons for imposing excise tax on soft drinks.
- The extent to which soft drink prices are affordable for consumers.

The third sections are considered the future-oriented tax awareness: A set of questions was designed for participants to indicate their level of agreement or disagreement using a five-point Likert scale: (Strongly Disagree—Disagree—Neutral—Agree—Strongly Agree), as follows:

- Knowledge that imposing excise tax on soft drinks will lead to a reduction in the quantities consumed.
- The prices of soft drinks at present are a motivator to seek healthy alternatives.
- The extent to which soft drinks will remain affordable for consumers in the future, given the continued imposition of the tax.
- Imposition of excise tax on soft drinks, and the consequent purchase of other healthier alternatives.
- The rate of increase in the consumption of soft drink alternatives will rise in the future.
- It is necessary for policymakers to impose taxes on goods that are harmful to health.

In developing this domain, we drew upon the study by Jalloun (25), which assessed participants' attitudes and behaviors regarding current taxes on soft drinks and energy drinks. However, in this domain, we focused on evaluating the attitudes and opinions of the sample members regarding the future imposition of excise tax.

Section 4 encompasses the anthropometric measures. The participants were requested to self-report their height and weight, which were utilized to compute body mass index (BMI) for the evaluation of their nutritional status. Given that the questionnaire aims to assess individual thoughts and attitudes, a five-point Likert scale was utilized (35). The participants' perceptions of awareness concerning taxation on SDs were evaluated by Yes/No questions. A five-point Likert scale (strongly disagree—disagree—neutral—agree—strongly agree) was employed to evaluate opinions (24).

The validation of questionnaires is an essential procedure in research to guarantee that the instrument accurately assesses its intended constructs and yields reliable, consistent outcomes. This questionnaire was distributed to nutritional specialists to evaluate its components and ensure it contained the necessary ingredients. A pilot survey was conducted on a small sample representative of the target group (20 individuals) to evaluate clarity, timeliness, reliability, and validity, and to adjust the questionnaire based on feedback.

The reliability of the instrument (questionnaire) is crucial to ensure that the results are consistent when administered to the same persons at different intervals. The aggregate outcome of the stability tool's scores and the stability scores for each dimension utilizing Cronbach's Alpha coefficient. Table 1 indicates that the stability coefficient of the study instrument (Cronbach's Alpha) varies between 0.752 and 0.841. The Cronbach's Alpha coefficients all surpass the minimal acceptable level of 0.7 (32, 33). Thus, we ascertain that the study instrument (questionnaire) demonstrates an adequate level of reliability, making it appropriate and useful for this research, and it can be dependent upon to achieve the study's objectives.

**Table 1:** The reliability outcomes of the questionnaire utilizing Cronbach's alpha.

Dimensions	No. of expressions	Cronbach's alpha coefficients
Awareness on SDs tax imposed	6	0.841
Future awareness on SDs tax imposed	6	0.752
Average		0.7965

### 2.3 Data analysis

The data was analyzed utilizing SPSS statistical software version 28. Descriptive analysis (frequencies and percentages) was employed to evaluate the demographic and socioeconomic characteristics of participants in Al-Ahsa, as well as their awareness of the imposition of excise taxes on SDs within the Al-Ahsa Governorate. The Chi Square Test was employed to evaluate the significance of the relationship between demographic and economic variables (education level and additional income) and the imposed tax on SDs since 2017. The Chi Square Test was utilized to evaluate the knowledge of implementing excise taxes on the consumption of SDs among participants in Al-Ahsa Governorate. The influence of demographic and economic variables (gender and age) on awareness of excise tax was evaluated using independent sample *T*-tests and ANOVA tables. The Spearman correlation was employed to evaluate the relationship between tax awareness and the reduction in the consumption of SDs due to the implementation of the excise tax. The link between tax awareness and the rise in the consumption of SDs among people of Al-Ahsa Governorate was evaluated using Spearman correlation. The association with the tax awareness on the BMI of participants in Al-Ahsa Governorate was evaluated using an ANOVA table. Furthermore, analysis of covariance (ANCOVA) was conducted to assess the association between awareness of the selective excise tax on soft drinks and participants' body mass index (BMI), adjusting for age, sex, marital status, educational level, and monthly household income.

## 3 Results and discussion

### 3.1 The demographic and socioeconomic characteristics of the participants

The demographic and socioeconomic attributes of the sample in the Al-Ahsa governorate were analyzed by descriptive statistics, emphasizing frequencies and percentages. Table 2 illustrates the distribution of participants in Al-Ahsa Governorate based on demographic and socioeconomic factors. Females constituted around 65.9% of the total, while men accounted for about 34.1%. Females demonstrate a heightened interest in nutritious diets relative to males. The data revealed that 49.7% of respondents are aged 20–30 years, 27.3% are under 20 years, and only 6.5% are above 41 years. The majority of participants were younger and below the productive age, and their nutritional security may be compromised by the consumption of soft drinks (SDs). Table 2 further illustrates the educational attainment of the individuals in Al-Ahsa. Most participants (70.8%) possess a university education, whereas only 9.5% have obtained postgraduate degrees. In contrast, merely 1.1% had completed middle school. The educational attainment figures reveal that most individuals are educated, which may be associated with their comprehension of the excise tax and the rationale for its imposition. Approximately 48.6% of the sample has a monthly salary between 5,000 and 10,000 SAR. Approximately 11.1% and 2.4% possess supplementary income within the ranges of ≥1,000–5,000 SAR and >5,000 SAR, respectively. The table depicts the distribution of SDs consumption, indicating that 86.2% of individuals engage in SDs consumption, whilst 13.8% do not. Thus, the importance of demographic and socioeconomics characteristics (age, gender, education, income, etc.) helps explain which individuals are aware of the tax, what they think about it, and whether they actually change their sugary drink consumption because of it (26).

**Table 2:** Distribution of the sample in Al-Ahsa Governorate according to their demographic and socioeconomic characteristics.

Participant characteristics	Categories	Frequency (%)
Sex	Female	244 (65.9%)
	Male	126 (34.1%)
Age	< 20	101 (27.3%)
	20–30	184 (49.7%)
	31–40	61 (16.5%)
	Older than 41	24 (6.5%)
Education level	Middle school	4 (1.1%)
	High school	69 (18.6%)
	University	262 (70.8%)
	Postgraduate	35 (9.5%)
Monthly income	>5,000–10,000 SAR	180 (48.6%)
	>10,000–15,000 SAR	50 (13.5%)
	> 15,000SAR	67 (18.1%)
Additional income	< 1,000 SAR	39 (10.5%)
	≥1,000–5,000 SAR	41 (11.1%)
	>5,000 SAR	9 (2.4%)
	Unstable income	9 (2.4%)
Do you drink SDs	Yes, I drink SDs	319 (86.2%)
	I do not drink SDs	51 (13.8%)

### 3.2 Awareness on the excise taxes on SDs

Table 3 illustrates the awareness of excise taxes levied on SDs among participants in Al-Ahsa Governorate. While 52.7% were unaware of the excise duty levied on SDs in the KSA since 2017, 47.3% were cognizant of the charge. The mean score for awareness of the excise duty on sugar-sweetened beverages (SDs) in KSA since 2017 was 1.47 on a 5-point Likert scale (1 = strongly disagree to 5 = strongly agree), indicating that, on average, participants tended to “strongly disagree” that they were aware of the excise duty on SDs. This suggests that most participants were not aware of the excise duty on SDs introduced in KSA in 2017. The table indicates that 64.9% are uninformed about the rationale behind the imposition of the excise tax on SDs in the KSA, whilst 35.1% are informed about the reasons for the tax's implementation. The limited awareness of the imposed tax may associate with the consumption of SDs. On the other hand, captures participants' self-reported change in consumption after the implementation of the excise tax. This measure reflects behavioral change attributed to the tax, as perceived and reported by respondents. Accordingly, approximately 75.4% indicated that the implementation of excise taxes did not influence their reduction in the consumption of SDs, whilst 24.6% of participants stated that the application of excise taxes did impact the decrease in SDs consumption. The weighted average of tax awareness across the sample participants was 1.35.

**Table 3:** The awareness on the excise taxes imposed on SDs among participants of the study in Al-Ahsa Governorate.

Variables	Responses		Average
Knowledge of the implementation of excise duty on SDs since 2017.	Yes	175 (47.3%)	1.47 (0.50)
	No	195 (52.7%)	
Knowledge of the rationale behind the implementation of an excise tax on SDs in the KSA.	Yes	130 (35.1%)	1.35 (0.47)
	No	240 (64.9%)	
The implementation of excise taxes has contributed to the reduction of SD consumption.	Yes	91 (24.6%)	1.25 (0.43)
	No	279 (75.4%)	
Weighted average.			1.35 (0.32)

Sample size = 370, The numbers in parentheses represents the percentages and the standard deviation.

### 3.3 Influence of socioeconomic factors on awareness of the excise tax

Table 4 illustrates the influence of demographic and economic variables on the awareness of taxes imposed on SDs since 2017. The results reveal that around 33.5% of participants with a university education were aware of the excise tax levied on SDs since 2017. Conversely, approximately 7% of the participants possess a higher level of education and are cognizant of the excise tax levied on SDs since 2017. The chi-square test indicated significant variations between educational level and the excise tax levied on SDs since 2017 at the 5% significance level. Thus, the individuals with higher education may be more aware of health risks and of the tax itself, so they are more likely to reduce SDs consumption or switch to healthier alternatives when the tax is introduced. This indicates that individuals with varying educational backgrounds react differently to the tax or adhere to it in distinct manners (27). This suggests that the behavioral reaction to excise taxes, such as decreased consumption of taxed harmful products, is more pronounced among people with higher education who possess a greater comprehension of health and price signals.

**Table 4:** The impact of some demographic and economic factors on tax imposed of the sample of Al-Ahsa governorate.

Variables	Categories	Imposing an excise tax on SDs since 2017		Chi-square	*P*-value
		Yes	No		
Educational level	Middle school	1 (0.3%)	3 (0.8%)	9.71^**^	0.02
	High school	26 (7.0%)	43 (11.6%)		
	University	124 (33.5%)	138 (37.3%)		
	Postgraduate	24 (6.5%)	11 (2.9%)		
Additional income (N-98)	< 1,000	16 (16.3%)	23 (23.5%)	9.61^**^	0.02
	≥1,000–5,000	26 (26.5%)	15 (15.3%)		
	>5,000	4 (4.1%)	5 (5.1%)		
	Unstable income	1 (1.0%)	8 (8.2%)		

Sample size = 370, ^**^indicate a statistically significant relationship at the 5% significance level.

Furthermore, approximately 26.5% of individuals with supplementary monthly income of 1,000–5,000 SAR were cognizant of the excise tax levied on SDs since 2017. Approximately 16.3% of individuals with an additional monthly income of < 1,000 SAR were cognizant of the excise tax levied on SDs since 2017. The Chi-square test results indicated significant differences between the additional income and excise tax levied on SDs since 2017 at the 5% significance level. This indicates that individuals with varying income levels react differently to the excise tax. It implies that the excise tax on SDs, implemented since 2017, has had differential effects across income groups, which supports the conclusion that income status modifies how the tax is experienced or responded to.

Table 5 illustrates the influence of certain demographic variables of participants on the awareness of excise taxes among residents of Al-Ahsa Governorate. The average tax awareness for males and females was approximately 1.41 and 1.32, respectively. The *t*-test results demonstrate a significant correlation between gender and excise tax awareness at the 5% significance level. Consequently, males exhibit greater average awareness ratings classified as “high awareness” in comparison to females. This means tax awareness is not uniform across genders, and this justified further discussion of gender-specific communication or education strategies regarding the tax. The table also depicts the average tax awareness score for those aged 20–30 years was 1.37, representing the largest participant category at 49.7%. The mean tax awareness score for those over 41 years of age was 1.43. The *F*-test results demonstrate a significant correlation between participants' ages and their average tax awareness scores at the 10% significance level. It means that the age is an important demographic factor in explaining variation in tax awareness, since the overall test indicates a statistically significant association at the 10% significance level. In comparison to the study conducted in Medina, Saudi Arabia, it was found that 56% of SD consumers endorsed the implementation of taxes, with the highest support among individuals aged 40–45 years, who possessed a university degree and were categorized as overweight or obese (25). Thirty-six percent of participants endorsed the introduction of a tax on packaged sugar-sweetened beverages, with the highest support shown among individuals aged 18–24 years (28). A poll conducted from 2015 to 2017 in demographically varied neighborhoods of the California Bay Area revealed differing levels of tax knowledge among people based on educational attainment, with lower awareness observed among individuals with minimal education. Views on the advantages of taxation differed based on educational level (23).

**Table 5:** The impact of specific demographic factors of participants on the awareness of selective tax implementation in Al-Ahsa governorate.

Variables	Categories		Average tax awareness	The value of the statistical test	*P*-value
Sex	Male	126 (34.0%)	1.41 (0.32)	2.61^**a^	0.01
	Female	244 (65.9%)	1.32 (0.32)		
Age	< 20	101 (27.3%)	1.28 (0.31)	2.56^*^b	0.05
	20–30	184 (49.7%)	1.37 (0.32)		
	31–40	61 (16.5%)	1.39 (0.31)		
	Older than 41	24 (6.5%)	1.43 (0.38)		

^a^*t*- test, ^b^ANOVA Test.

Sample size = 370, The number in parentheses represents the standard deviation, ^*^, ^**^ indicate a statistically significant relationship at the 10% and 5% significance level, respectively.

Table 6 illustrates the relationship between awareness on the excise taxes levied and consumption of SDs among participants in Al-Ahsa Governorate. The table presents the current consumption status refers to whether participants reported consuming soft drinks at the time of the survey, irrespective of any changes over time. It reflects a snapshot of behavior at the point of data collection. If they consume or not consumed as reported by respondents. The findings indicate that approximately 55.7% of those who consumed SDs lack understanding of the excise tax, whereas only 30.5% of SD consumers are aware of the imposed excise tax. Conversely, around 4.3% of individuals who did not consume SDs possess understanding of the excise duty. The chi-square result reveals no significant association between participants' tax awareness and their intake of soft drinks (SDs). Even though an excise tax has been in place on soft drinks (SDs) since 2017, participants' level of awareness about this tax did not significantly influence whether or how much they consumed soft drinks. Thus, awareness alone was not enough to alter behavior, despite the tax being implemented. Thus, means other factors (e.g., habits, taste preferences, income, availability of alternatives) may be more influential than tax awareness in determining consumption.

**Table 6:** The relationship between awareness on the implementation of excise taxes and consumption of SDs among participants in Al-Ahsa Governorate.

Variables		Tax awareness		Chi-Square	*P*-value
		No	Yes		
Drink SDs	No	35 (9.4%)	16 (4.3%)	0.31	0.57
	Yes	206 (55.7%)	113 (30.5%)		

Sample size = 370, numbers in parentheses represent percentage.

### 3.4 Awareness on excise tax and alternatives of SDs

Table 7 displays the results of the Spearman connection between awareness of the imposed tax and the reduction in SDs consumption due to the excise tax implemented. Participants aware of the excise duty levied on SDs since 2017 have reduced their intake of SDs. The Spearman coefficient results demonstrate a statistically significant link at the 1% level between the reduction in SDs consumption due to the imposed excise tax and awareness of the excise tax on SDs since 2017. The understanding of the rationale behind the introduction of an excise tax on SDs is correlated with a reduction in the consumption of SDs. The Spearman coefficient demonstrates statistical significance at the 5% level. It means that people who know about the excise tax on sugary drinks (SDs) are more likely to cut down their consumption of these drinks, and this link is statistically significant, suggesting that this signaling works better when people are aware of the tax awareness amplifies the association of the excise tax with reducing sugary-drink intake. The results align with the research by (29), which indicated that the SDs tax altered consumption patterns, as the proportion of participants consuming SDs post-tax reduced by 19% relative to the pre-tax scenario. The study by Jalloun (25) demonstrated that a significant proportion of participants indicated a reduction in the consumption of SDs following the imposition of taxes. Nevertheless, this research evaluated participants' attitudes and practices about taxes levied on the use of SDs without examining tax awareness or the rationale for the implementation of these taxes.

**Table 7:** The relationship between tax awareness and the reduction in the consumption of SDs due to the imposed excise tax.

Correlation	Knowledge of the implementation of excise duty on SDs since 2017	Knowledge of the rationale behind the implementation of an excise tax on SDs in the KSA
Sperman correlation	0.213^***^	0.119^**^
*P*-value	0.000	0.02

Sample size = 370, ^***^, ^**^ indicate a statistically significant relationship at the 1% and 5% significance level, respectively.

Table 8 illustrates the association between awareness of excise taxes and the reduction in consumption of SDs, as well as the rate of increase in the consumption of SD alternatives. The results of the Spearman correlation coefficient reveal a relationship between the increase in consumption of SD alternatives and the decrease in consumption of SDs due to imposed excise taxes. The Spearman coefficient (0.117) signifies a positive and weak connection, with statistical significance at the 5% level. This indicates that individuals' understanding of the tax is statistically correlated with their reduction of sugar-sweetened drinks and their transition to other beverages such as water, milk, or unsweetened drinks. Consequently, increased awareness of the excise tax correlates with a more significant reduction in SD consumption; those informed about the tax and its intent are more inclined to decrease their consumption of SDs. This outcome aligns with (25) findings, which indicated that 69% of the study group reported that taxes on SDs and EDs impeded their usage of these products. Sixty percent of interviewees indicated that they used alternative beverages as a result of levies levied on sugary drinks and energy drinks. Moreover, 89% of study participants assert that the government ought to impose taxes on foods and beverages detrimental to health in Medina, Saudi Arabia. Royo-Bordonada et al. (30) reported that following the tax implementation, the prevalence of consumers of untaxed beverages rose by 7.4% in Madrid and 4% in Barcelona.

**Table 8:** The correlation between tax awareness and the rising consumption of SDs alternatives among participants in Al-Ahsa governorate.

Correlation	Tax awareness and rising consumption of SD alternatives
Sperman correlation	0.117^**^
*P*-value	0.02

Sample size = 370, ^**^ indicate a statistically significant relationship at the 5% significance level.

### 3.5 Impact of awareness on the excise tax related to body mass index (BMI)

Table 9 illustrates the distribution of participants in Al-Ahsa Governorate according on BMI. The results indicate that 47.6% of participants have a normal weight, 32.2% are overweight, and just 17% are classified as obese )24) To examine the association between excise tax awareness and BMI, we first conducted a one-way ANOVA. Because this model does not adjust for potential confounders. Table 9 illustrates the association of tax awareness on BMI by an ANOVA analysis. The *F*-test results demonstrate a strong association between BMI and tax awareness at a 5% statistical level. The results indicate that awareness of taxation related to the nutritional status of the individuals. This implies if the awareness is high, people may reduce intake of taxed, less healthy foods such as sugar-sweetened beverages and hence shift toward healthier, untaxed or subsidized options, improving overall diet quality and potentially weight status. Consequently, imposing the tax on SDs will be associate with improving with nutritional status and health. Jalloun and Qurban (29) established that the average weight prior to the tax exceeded that after to its implementation by 4.39 kilos among SDs consumers and by approximately 0.45 g among non-consumers. Weight was markedly correlated with the use of SDs compared to their non-consumption. The danger of weight gain associated with sugar present in many beverages, including soft drinks and energy drinks, has been demonstrated in numerous longitudinal investigations and clinical trials. Excessive sugar intake, especially from sugary beverages, significantly contributes to the obesity epidemic (31). Rivard et al. (28) indicated that 36% of participants supported the imposition of a tax on packaged sugar-sweetened beverages (SSBs), with the highest endorsement shown among individuals with a BMI over 30 kg/m^2^. Consequently, it may be inferred that SD taxes influence food habits, and these behavioral modifications are substantial enough to result in considerable alterations in body weight.

**Table 9:** The impact of tax awareness on the BMI of participants in Al-Ahsa governorate.

Variables	Weighted average of tax awareness		*F*-test	*P*-Value
Underweight	45 (12.2%)	1.40 (0.28)	3.17^**^	0.02
Normal	176 (47.6%)	1.30 (0.32)		
Overweight	86 (23.2%)	1.39 (0.32)		
Obesity	63 (17.0%)	1.42 (0.35)	

Sample size = 370, The number in parentheses represents the standard deviation, ^**^indicate a statistically significant relationship at the 5% significance level.

The analysis of covariance (ANCOVA) was used to evaluate the association between awareness of the selective excise tax on soft drinks and BMI controlling for age, sex, marital status, educational attainment, and monthly household income. The findings of the analysis of covariance (ANCOVA) on the association between awareness of selective excise taxes and categories of body mass index (BMI) are presented in Table 10 with adjustment for chosen demographic, social and economic variables. Results of ANCOVA showed that, controlling for age, sex, marital status, educational level and monthly household income, there was no significant association between awareness of the selective excise tax on soft drinks and BMI categories [*F*(1, 363) = 0.03, *p* = 0.86, η^2^*p* = 0.00]. In other words, awareness or unawareness of the tax did not produce significant BMI variations when controlling for other variables.

**Table 10:** Results of the analysis of covariance (ANCOVA) examining the association between awareness of selective excise taxes and body mass index (BMI) categories, adjusted for selected demographic, social, and economic variables.

Source	Type III sum of squares	df	Mean square	*F*	*P*-value	Partial eta squared (η^2^*p*)
Corrected model	58.126	6	9.688	14.095	0.000	0.189
Intercept	47.723	1	47.723	69.433	0.000	0.161
Age	14.847	1	14.847	21.601	0.000	0.056
Sex	8.797	1	8.797	12.799	0.000	0.034
Marital status	2.767	1	2.767	4.026	0.046	0.011
Educational level	1.040	1	1.040	1.513	0.219	0.004
Monthly household income	0.709	1	0.709	1.031	0.311	0.003
Tax awareness	0.021	1	0.021	0.031	0.860	0.000
Error	249.498	363	0.687	–	–	–
corrected total	307.624	369	–	–	–	–

Dependent variable = BMI categories. *R*^2^ = 0.189, Adjusted *R*^2^ = 0.176.

BMI, on the other hand, was substantially associated with various control variables. Age was strongly positively associated [*F*(1, 363) = 21.60, *p* < 0.001, η^2^*p* = 0.056], with older people tending to be in higher BMI categories. Sex was also significant [*F*(1, 363) = 12.80, *p* < 0.001, η^2^*p* = 0.034] indicating that BMI distribution was different between males and females. Marital status had a lesser but still significant influence [*F*(1, 363) = 4.03, *p* = 0.046, η^2^*p* = 0.011]. In this model, educational level and household income were not significantly related with BMI (*p* > 0.05). The whole model explained 18.9% of the variation of BMI categories (*R*^2^ = 0.189) while tax awareness contributed almost nil to this explanation. This finding implies that awareness of the excise tax is not related to lower or higher BMI in the context of controlling demographic and socioeconomic variables for this population.

Although the unadjusted analysis showed a statistically significant difference in BMI according to excise-tax awareness, this association disappeared after controlling demographic and socioeconomic characteristics. These findings suggest that the initial relationship may have been attributable to differences in the participants' background characteristics rather than to tax awareness itself. Thus, excise-tax awareness was not independently associated with BMI in the adjusted analysis.

## 4 Conclusion

This study examined the relationship between socioeconomic determinants, awareness of the excise tax on soft drinks consumption behaviors, and nutritional status among adults in Al-Ahsa Province, Saudi Arabia. The findings reveal that awareness of the excise tax remains relatively low despite its implementation in 2017, with more than half of the participants unaware of the tax and its underlying health rationale. Socioeconomic factors, particularly education level, income, gender, and age, were significantly associated with tax awareness, indicating disparities in access to health and fiscal policy information. Although awareness alone did not significantly determine whether individuals consumed soft drinks, it played a critical role in shaping behavioral responses. Participants with higher tax awareness were more likely to reduce soft drink consumption, shift toward healthier beverage alternatives, and exhibit more favorable BMI classifications. Although the one-way ANOVA showed a significant association between excise-tax awareness and BMI, this association was not significant after adjusting for demographic and socioeconomic factors in the ANCOVA. Thus, excise-tax awareness was not independently associated with BMI in this population. Thus, policy and public health measures to address obesity should go beyond awareness of the tax to better behavioral, environmental, and communication strategies, and future studies should examine more direct linkages between taxation, consumption, and weight consequences. Furthermore, the findings suggest that excise taxation can contribute to improved dietary behaviors and nutritional outcomes when accompanied by effective public awareness and education strategies. Simply imposing a tax may be insufficient to achieve optimal public health impact without ensuring that individuals understand its purpose and benefits. This suggests that policy measures may need to go beyond taxation and awareness, such as stronger health education campaigns, improving access to healthy alternatives, or additional regulatory actions. Thus, future studies should adopt longitudinal or interventional designs to better examine the causal relationship between excise tax awareness, soft drink consumption, and changes in nutritional status over time. The use of objectively measured anthropometric data is recommended to reduce potential reporting bias. Expanding research to include different age groups and regions of Saudi Arabia would improve the generalizability of findings. Additionally, evaluating the effectiveness of targeted public awareness and education campaigns may help to enhance the impact of excise taxation as a public health strategy.

## Data Availability

The original contributions presented in the study are included in the article/supplementary material, further inquiries can be directed to the corresponding author.
